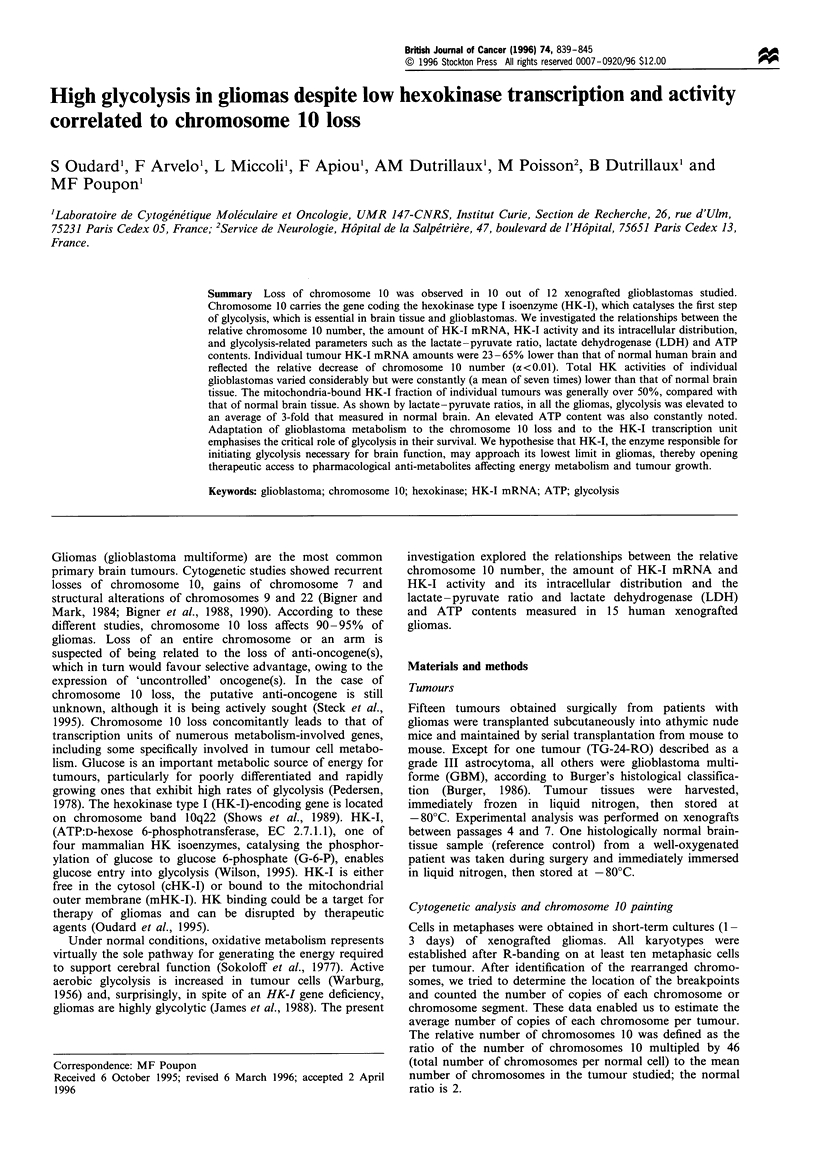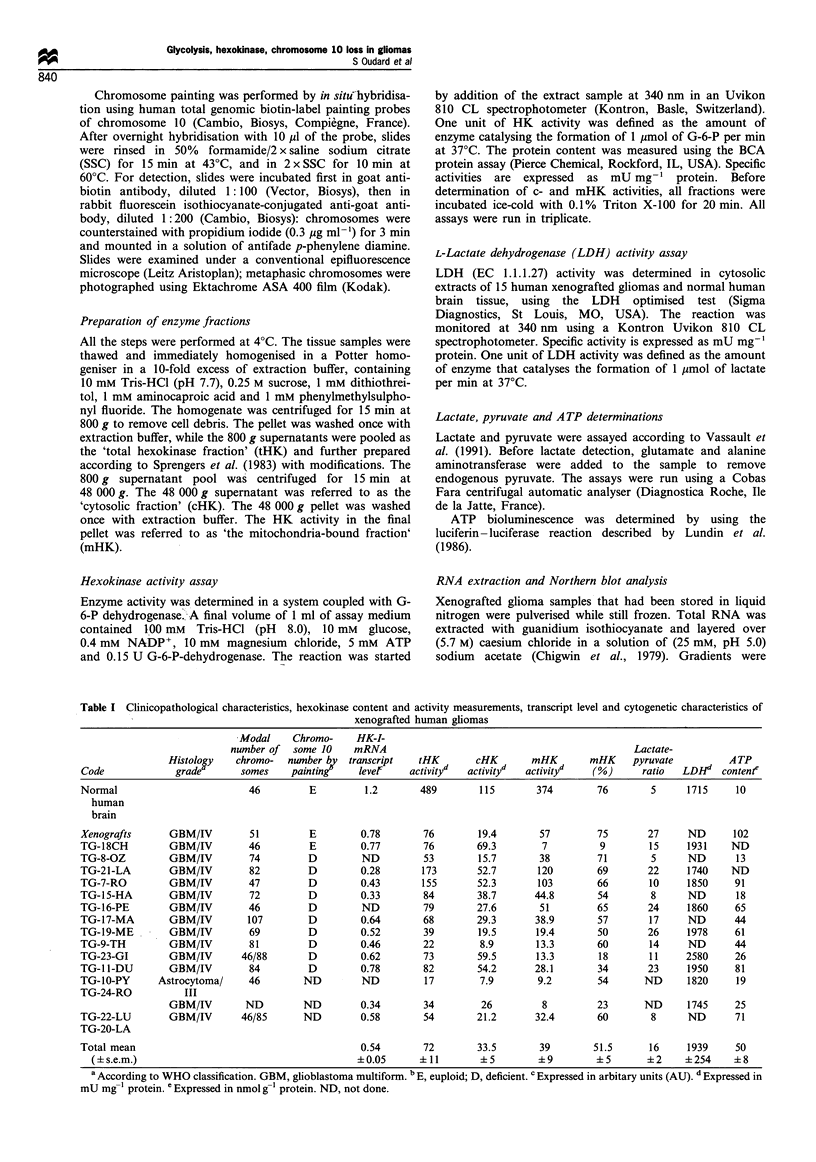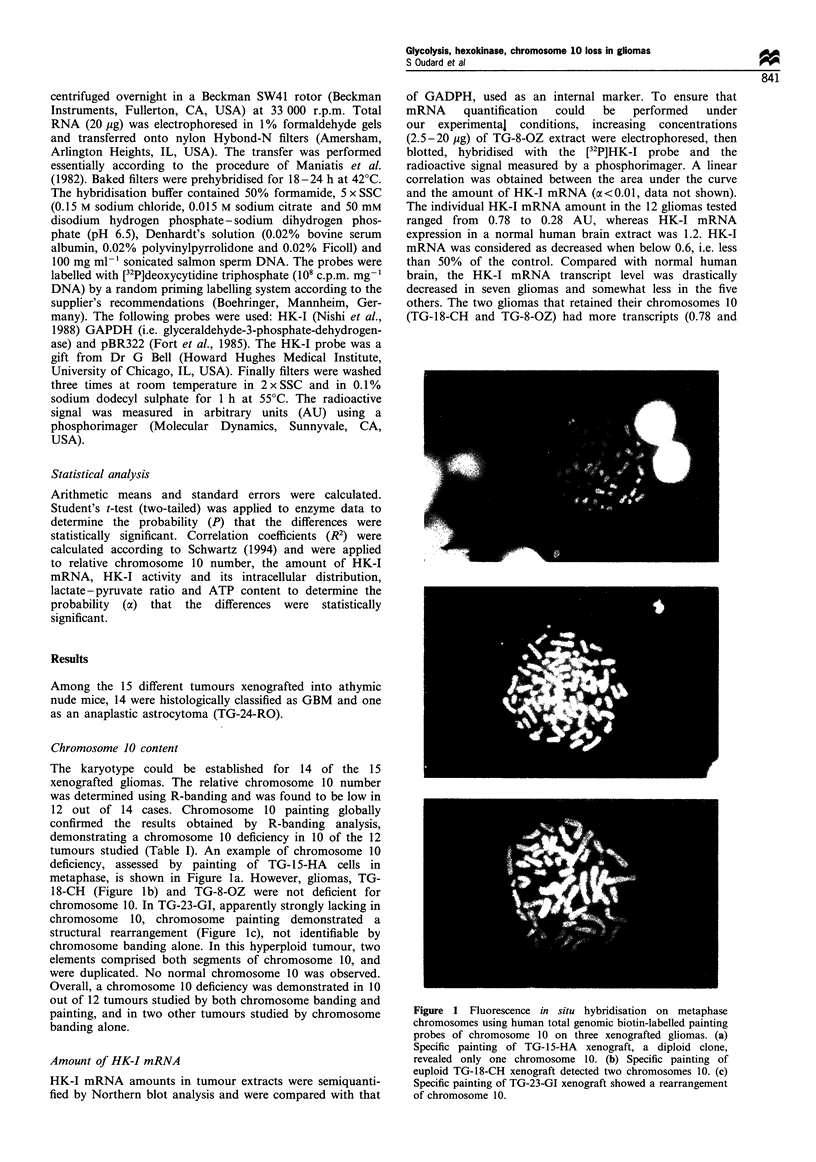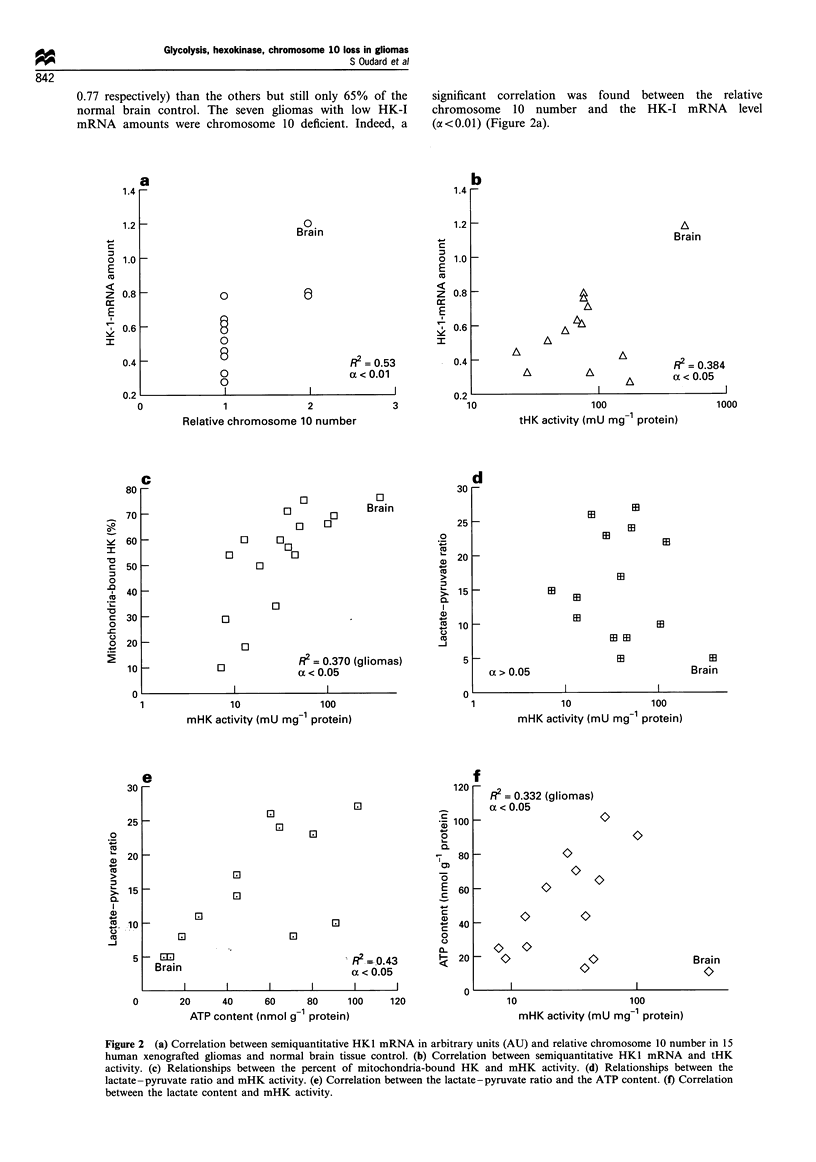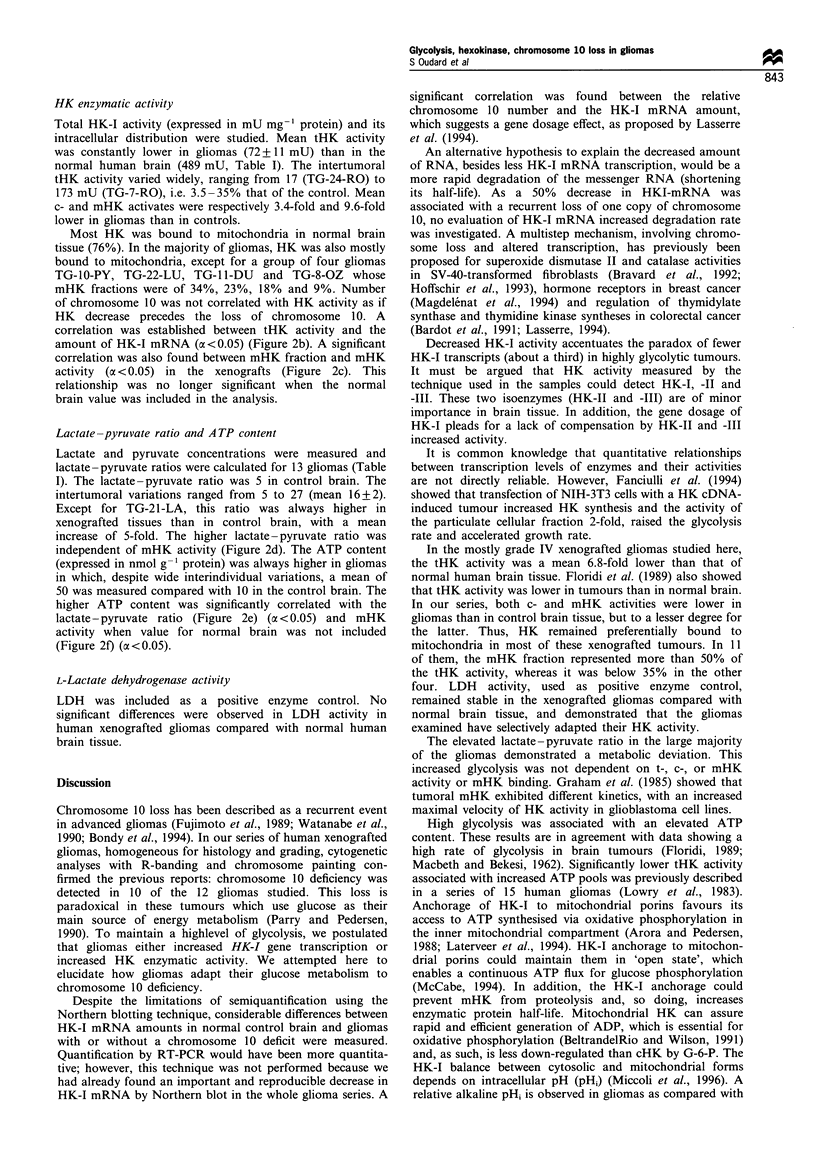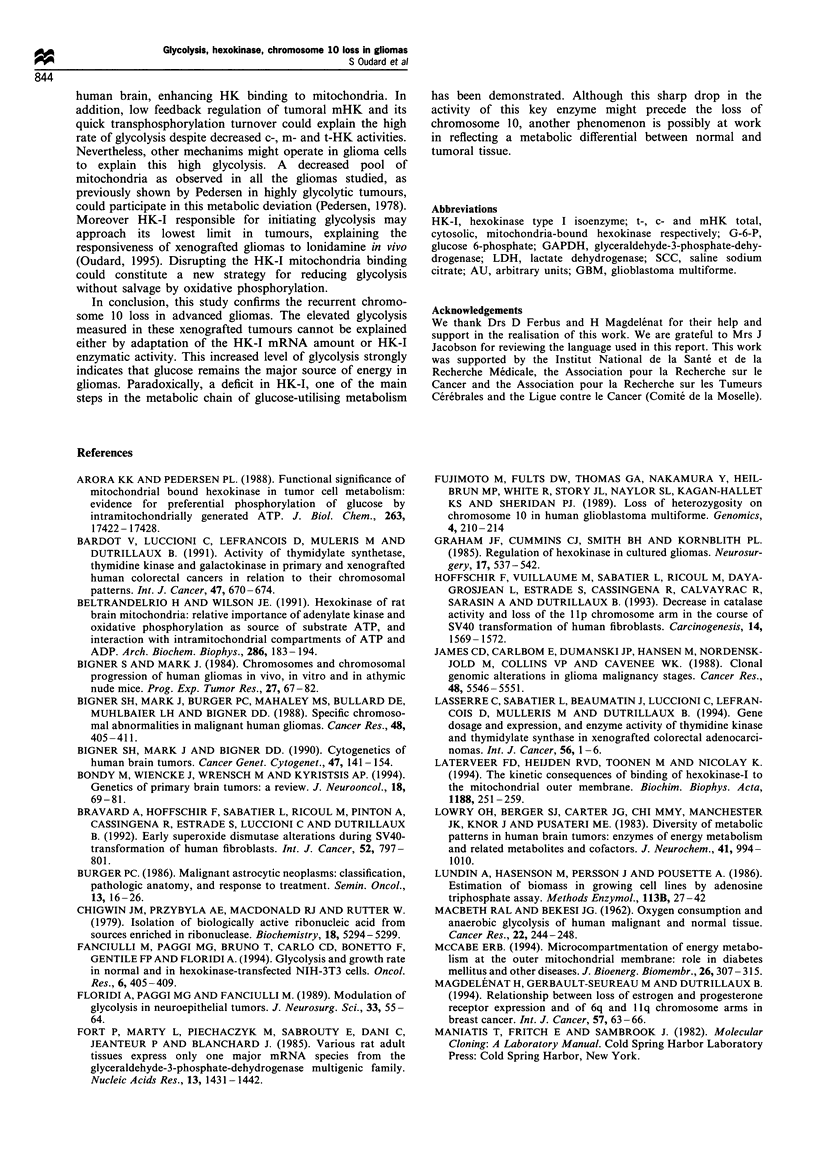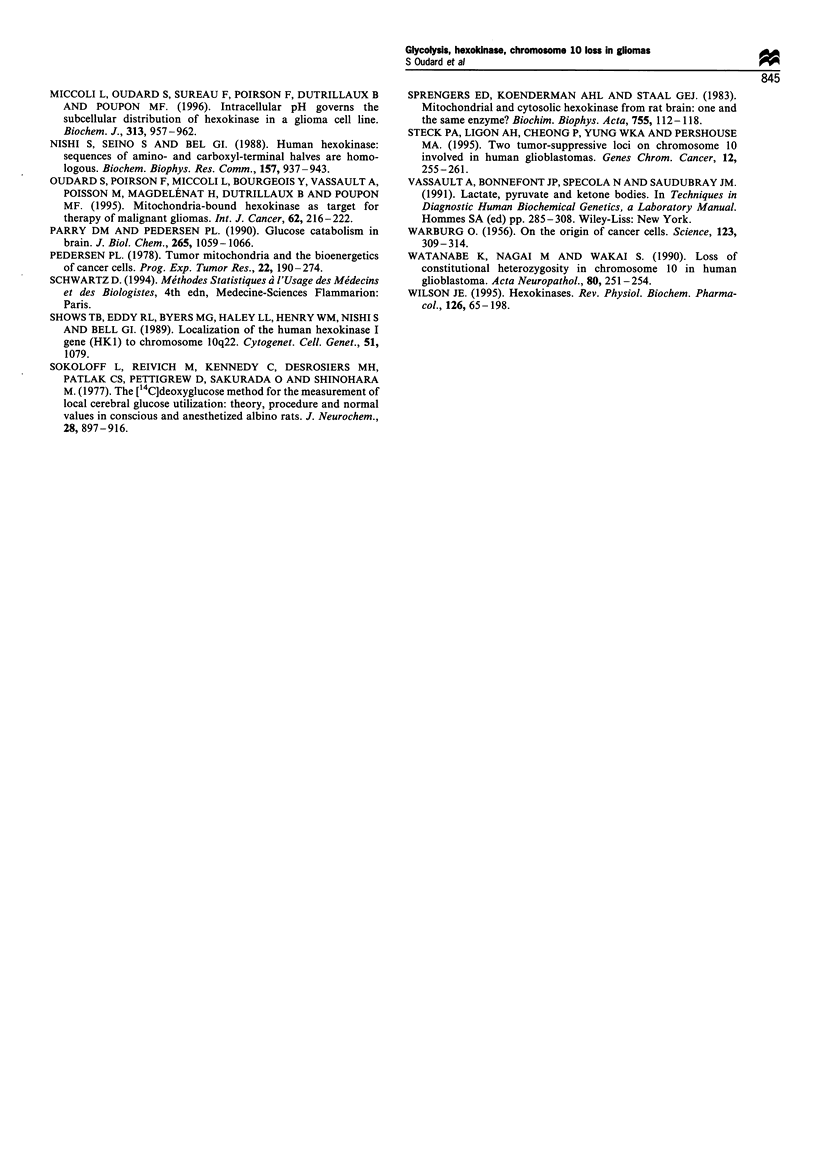# High glycolysis in gliomas despite low hexokinase transcription and activity correlated to chromosome 10 loss.

**DOI:** 10.1038/bjc.1996.446

**Published:** 1996-09

**Authors:** S. Oudard, F. Arvelo, L. Miccoli, F. Apiou, A. M. Dutrillaux, M. Poisson, B. Dutrillaux, M. F. Poupon

**Affiliations:** Laboratoire de Cytogénétique Moléculaire et Oncologie, UMR 147-CNRS, Paris, France.

## Abstract

**Images:**


British Journal of Cancer (1996) 74, 839-845
© 1996 Stockton Press All rights reserved 0007-0920/96 $12.00

### High glycolysis in gliomas despite low hexokinase transcription and activity correlated to chromosome 10 loss

S Oudard[1], F Arvelo[1], L Miccoli[1], F Apiou[1], AM Dutrillaux[1], M Poisson[2], B Dutrillaux[1] and MF Poupon[1]

[1]*Laboratoire de Cytogénétique Moléculaire et Oncologie, UMR 147-CNRS, Institut Curie, Section de Recherche, 26, rue d'Ulm, 75231 Paris Cedex 05, France;* [2]*Service de Neurologie, Hôpital de la Salpêtrière, 47, boulevard de l'Hôpital, 75651 Paris Cedex 13, France.*

**Summary** Loss of chromosome 10 was observed in 10 out of 12 xenografted glioblastomas studied. Chromosome 10 carries the gene coding the hexokinase type I isoenzyme (HK-I), which catalyses the first step of glycolysis, which is essential in brain tissue and glioblastomas. We investigated the relationships between the relative chromosome 10 number, the amount of HK-I mRNA, HK-I activity and its intracellular distribution, and glycolysis-related parameters such as the lactate–pyruvate ratio, lactate dehydrogenase (LDH) and ATP contents. Individual tumour HK-I mRNA amounts were 23–65% lower than that of normal human brain and reflected the relative decrease of chromosome 10 number ($\alpha < 0.01$). Total HK activities of individual glioblastomas varied considerably but were constantly (a mean of seven times) lower than that of normal brain tissue. The mitochondria-bound HK-I fraction of individual tumours was generally over 50%, compared with that of normal brain tissue. As shown by lactate–pyruvate ratios, in all the gliomas, glycolysis was elevated to an average of 3-fold that measured in normal brain. An elevated ATP content was also constantly noted. Adaptation of glioblastoma metabolism to the chromosome 10 loss and to the HK-I transcription unit emphasises the critical role of glycolysis in their survival. We hypothesise that HK-I, the enzyme responsible for initiating glycolysis necessary for brain function, may approach its lowest limit in gliomas, thereby opening therapeutic access to pharmacological anti-metabolites affecting energy metabolism and tumour growth.

**Keywords:** glioblastoma; chromosome 10; hexokinase; HK-I mRNA; ATP; glycolysis

Gliomas (glioblastoma multiforme) are the most common primary brain tumours. Cytogenetic studies showed recurrent losses of chromosome 10, gains of chromosome 7 and structural alterations of chromosomes 9 and 22 (Bigner and Mark, 1984; Bigner *et al.*, 1988, 1990). According to these different studies, chromosome 10 loss affects 90–95% of gliomas. Loss of an entire chromosome or an arm is suspected of being related to the loss of anti-oncogene(s), which in turn would favour selective advantage, owing to the expression of 'uncontrolled' oncogene(s). In the case of chromosome 10 loss, the putative anti-oncogene is still unknown, although it is being actively sought (Steck *et al.*, 1995). Chromosome 10 loss concomitantly leads to that of transcription units of numerous metabolism-involved genes, including some specifically involved in tumour cell metabolism. Glucose is an important metabolic source of energy for tumours, particularly for poorly differentiated and rapidly growing ones that exhibit high rates of glycolysis (Pedersen, 1978). The hexokinase type I (HK-I)-encoding gene is located on chromosome band 10q22 (Shows *et al.*, 1989). HK-I, (ATP:D-hexose 6-phosphotransferase, EC 2.7.1.1), one of four mammalian HK isoenzymes, catalysing the phosphorylation of glucose to glucose 6-phosphate (G-6-P), enables glucose entry into glycolysis (Wilson, 1995). HK-I is either free in the cytosol (cHK-I) or bound to the mitochondrial outer membrane (mHK-I). HK binding could be a target for therapy of gliomas and can be disrupted by therapeutic agents (Oudard *et al.*, 1995).

Under normal conditions, oxidative metabolism represents virtually the sole pathway for generating the energy required to support cerebral function (Sokoloff *et al.*, 1977). Active aerobic glycolysis is increased in tumour cells (Warburg, 1956) and, surprisingly, in spite of an *HK-I* gene deficiency, gliomas are highly glycolytic (James *et al.*, 1988). The present investigation explored the relationships between the relative chromosome 10 number, the amount of HK-I mRNA and HK-I activity and its intracellular distribution and the lactate–pyruvate ratio and lactate dehydrogenase (LDH) and ATP contents measured in 15 human xenografted gliomas.

Correspondence: MF Poupon
Received 6 October 1995; revised 6 March 1996; accepted 2 April 1996

### Materials and methods

### *Tumours*

Fifteen tumours obtained surgically from patients with gliomas were transplanted subcutaneously into athymic nude mice and maintained by serial transplantation from mouse to mouse. Except for one tumour (TG-24-RO) described as a grade III astrocytoma, all others were glioblastoma multiforme (GBM), according to Burger's histological classification (Burger, 1986). Tumour tissues were harvested, immediately frozen in liquid nitrogen, then stored at −80°C. Experimental analysis was performed on xenografts between passages 4 and 7. One histologically normal brain-tissue sample (reference control) from a well-oxygenated patient was taken during surgery and immediately immersed in liquid nitrogen, then stored at −80°C.

### *Cytogenetic analysis and chromosome 10 painting*

Cells in metaphases were obtained in short-term cultures (1–3 days) of xenografted gliomas. All karyotypes were established after R-banding on at least ten metaphasic cells per tumour. After identification of the rearranged chromosomes, we tried to determine the location of the breakpoints and counted the number of copies of each chromosome or chromosome segment. These data enabled us to estimate the average number of copies of each chromosome per tumour. The relative number of chromosomes 10 was defined as the ratio of the number of chromosomes 10 multipled by 46 (total number of chromosomes per normal cell) to the mean number of chromosomes in the tumour studied; the normal ratio is 2.

Chromosome painting was performed by *in situ* hybridisation using human total genomic biotin-label painting probes of chromosome 10 (Cambio, Biosys, Compiègne, France). After overnight hybridisation with 10 $\mu$l of the probe, slides were rinsed in 50% formamide/2×saline sodium citrate (SSC) for 15 min at 43°C, and in 2×SSC for 10 min at 60°C. For detection, slides were incubated first in goat anti-biotin antibody, diluted 1:100 (Vector, Biosys), then in rabbit fluorescein isothiocyanate-conjugated anti-goat antibody, diluted 1:200 (Cambio, Biosys): chromosomes were counterstained with propidium iodide (0.3 $\mu$g $ml^{-1}$) for 3 min and mounted in a solution of antifade *p*-phenylene diamine. Slides were examined under a conventional epifluorescence microscope (Leitz Aristoplan); metaphasic chromosomes were photographed using Ektachrome ASA 400 film (Kodak).

### *Preparation of enzyme fractions*

All the steps were performed at 4°C. The tissue samples were thawed and immediately homogenised in a Potter homogeniser in a 10-fold excess of extraction buffer, containing 10 mM Tris-HCl (pH 7.7), 0.25 M sucrose, 1 mM dithiothreitol, 1 mM aminocaproic acid and 1 mM phenylmethylsulphonyl fluoride. The homogenate was centrifuged for 15 min at 800 *g* to remove cell debris. The pellet was washed once with extraction buffer, while the 800 *g* supernatants were pooled as the 'total hexokinase fraction' (tHK) and further prepared according to Sprengers *et al.* (1983) with modifications. The 800 *g* supernatant pool was centrifuged for 15 min at 48 000 *g*. The 48 000 *g* supernatant was referred to as the 'cytosolic fraction' (cHK). The 48 000 *g* pellet was washed once with extraction buffer. The HK activity in the final pellet was referred to as 'the mitochondria-bound fraction' (mHK).

### *Hexokinase activity assay*

Enzyme activity was determined in a system coupled with G-6-P dehydrogenase. A final volume of 1 ml of assay medium contained 100 mM Tris-HCl (pH 8.0), 10 mM glucose, 0.4 mM $NADP^+$, 10 mM magnesium chloride, 5 mM ATP and 0.15 U G-6-P-dehydrogenase. The reaction was started by addition of the extract sample at 340 nm in an Uvikon 810 CL spectrophotometer (Kontron, Basle, Switzerland). One unit of HK activity was defined as the amount of enzyme catalysing the formation of 1 $\mu$mol of G-6-P per min at 37°C. The protein content was measured using the BCA protein assay (Pierce Chemical, Rockford, IL, USA). Specific activities are expressed as mU $mg^{-1}$ protein. Before determination of c- and mHK activities, all fractions were incubated ice-cold with 0.1% Triton X-100 for 20 min. All assays were run in triplicate.

### *L-Lactate dehydrogenase (LDH) activity assay*

LDH (EC 1.1.1.27) activity was determined in cytosolic extracts of 15 human xenografted gliomas and normal human brain tissue, using the LDH optimised test (Sigma Diagnostics, St Louis, MO, USA). The reaction was monitored at 340 nm using a Kontron Uvikon 810 CL spectrophotometer. Specific activity is expressed as mU $mg^{-1}$ protein. One unit of LDH activity was defined as the amount of enzyme that catalyses the formation of 1 $\mu$mol of lactate per min at 37°C.

### *Lactate, pyruvate and ATP determinations*

Lactate and pyruvate were assayed according to Vassault *et al.* (1991). Before lactate detection, glutamate and alanine aminotransferase were added to the sample to remove endogenous pyruvate. The assays were run using a Cobas Fara centrifugal automatic analyser (Diagnostica Roche, Ile de la Jatte, France).

ATP bioluminescence was determined by using the luciferin–luciferase reaction described by Lundin *et al.* (1986).

### *RNA extraction and Northern blot analysis*

Xenografted glioma samples that had been stored in liquid nitrogen were pulverised while still frozen. Total RNA was extracted with guanidium isothiocyanate and layered over (5.7 M) caesium chloride in a solution of (25 mM, pH 5.0) sodium acetate (Chigwin *et al.*, 1979). Gradients were

**Table I** Clinicopathological characteristics, hexokinase content and activity measurements, transcript level and cytogenetic characteristics of xenografted human gliomas

| Code | *Histology grade*[a] | *Modal number of chromosomes* | *Chromosome 10 number by painting*[b] | *HK-I-mRNA transcript level*[c] | *tHK activity*[d] | *cHK activity*[d] | *mHK activity*[d] | *mHK (%)* | *Lactate-pyruvate ratio* | *LDH*[d] | *ATP content*[e] |
|---|---|---|---|---|---|---|---|---|---|---|---|
| Normal human brain | | 46 | E | 1.2 | 489 | 115 | 374 | 76 | 5 | 1715 | 10 |
| *Xenografts* | GBM/IV | 51 | E | 0.78 | 76 | 19.4 | 57 | 75 | 27 | ND | 102 |
| TG-18CH | GBM/IV | 46 | E | 0.77 | 76 | 69.3 | 7 | 9 | 15 | 1931 | ND |
| TG-8-OZ | GBM/IV | 74 | D | ND | 53 | 15.7 | 38 | 71 | 5 | ND | 13 |
| TG-21-LA | GBM/IV | 82 | D | 0.28 | 173 | 52.7 | 120 | 69 | 22 | 1740 | ND |
| TG-7-RO | GBM/IV | 47 | D | 0.43 | 155 | 52.3 | 103 | 66 | 10 | 1850 | 91 |
| TG-15-HA | GBM/IV | 72 | D | 0.33 | 84 | 38.7 | 44.8 | 54 | 8 | ND | 18 |
| TG-16-PE | GBM/IV | 46 | D | ND | 79 | 27.6 | 51 | 65 | 24 | 1860 | 65 |
| TG-17-MA | GBM/IV | 107 | D | 0.64 | 68 | 29.3 | 38.9 | 57 | 17 | ND | 44 |
| TG-19-ME | GBM/IV | 69 | D | 0.52 | 39 | 19.5 | 19.4 | 50 | 26 | 1978 | 61 |
| TG-9-TH | GBM/IV | 81 | D | 0.46 | 22 | 8.9 | 13.3 | 60 | 14 | ND | 44 |
| TG-23-GI | GBM/IV | 46/88 | D | 0.62 | 73 | 59.5 | 13.3 | 18 | 11 | 2580 | 26 |
| TG-11-DU | GBM/IV | 84 | D | 0.78 | 82 | 54.2 | 28.1 | 34 | 23 | 1950 | 81 |
| TG-10-PY | Astrocytoma/ | 46 | ND | ND | 17 | 7.9 | 9.2 | 54 | ND | 1820 | 19 |
| TG-24-RO | III | | | | | | | | | | |
| | GBM/IV | ND | ND | 0.34 | 34 | 26 | 8 | 23 | ND | 1745 | 25 |
| TG-22-LU | GBM/IV | 46/85 | ND | 0.58 | 54 | 21.2 | 32.4 | 60 | 8 | ND | 71 |
| TG-20-LA | | | | | | | | | | | |
| Total mean (±s.e.m.) | | | | 0.54 ±0.05 | 72 ±11 | 33.5 ±5 | 39 ±9 | 51.5 ±5 | 16 ±2 | 1939 ±254 | 50 ±8 |

[a] According to WHO classification. GBM, glioblastoma multiform. [b] E, euploid; D, deficient. [c] Expressed in arbitary units (AU). [d] Expressed in mU $mg^{-1}$ protein. [e] Expressed in nmol $g^{-1}$ protein. ND, not done.

centrifuged overnight in a Beckman SW41 rotor (Beckman Instruments, Fullerton, CA, USA) at 33 000 r.p.m. Total RNA (20 $\mu$g) was electrophoresed in 1% formaldehyde gels and transferred onto nylon Hybond-N filters (Amersham, Arlington Heights, IL, USA). The transfer was performed essentially according to the procedure of Maniatis *et al.* (1982). Baked filters were prehybridised for 18–24 h at 42°C. The hybridisation buffer contained 50% formamide, 5 × SSC (0.15 M sodium chloride, 0.015 M sodium citrate and 50 mM disodium hydrogen phosphate–sodium dihydrogen phosphate (pH 6.5), Denhardt's solution (0.02% bovine serum albumin, 0.02% polyvinylpyrrolidone and 0.02% Ficoll) and 100 mg ml$^{-1}$ sonicated salmon sperm DNA. The probes were labelled with [$^{32}$P]deoxycytidine triphosphate (10$^8$ c.p.m. mg$^{-1}$ DNA) by a random priming labelling system according to the supplier's recommendations (Boehringer, Mannheim, Germany). The following probes were used: HK-I (Nishi *et al.*, 1988) GAPDH (i.e. glyceraldehyde-3-phosphate-dehydrogenase) and pBR322 (Fort *et al.*, 1985). The HK-I probe was a gift from Dr G Bell (Howard Hughes Medical Institute, University of Chicago, IL, USA). Finally filters were washed three times at room temperature in 2 × SSC and in 0.1% sodium dodecyl sulphate for 1 h at 55°C. The radioactive signal was measured in arbitrary units (AU) using a phosphorimager (Molecular Dynamics, Sunnyvale, CA, USA).

*Statistical analysis*

Arithmetic means and standard errors were calculated. Student's *t*-test (two-tailed) was applied to enzyme data to determine the probability (*P*) that the differences were statistically significant. Correlation coefficients ($R^2$) were calculated according to Schwartz (1994) and were applied to relative chromosome 10 number, the amount of HK-I mRNA, HK-I activity and its intracellular distribution, lactate–pyruvate ratio and ATP content to determine the probability ($\alpha$) that the differences were statistically significant.

### Results

Among the 15 different tumours xenografted into athymic nude mice, 14 were histologically classified as GBM and one as an anaplastic astrocytoma (TG-24-RO).

*Chromosome 10 content*

The karyotype could be established for 14 of the 15 xenografted gliomas. The relative chromosome 10 number was determined using R-banding and was found to be low in 12 out of 14 cases. Chromosome 10 painting globally confirmed the results obtained by R-banding analysis, demonstrating a chromosome 10 deficiency in 10 of the 12 tumours studied (Table I). An example of chromosome 10 deficiency, assessed by painting of TG-15-HA cells in metaphase, is shown in Figure 1a. However, gliomas, TG-18-CH (Figure 1b) and TG-8-OZ were not deficient for chromosome 10. In TG-23-GI, apparently strongly lacking in chromosome 10, chromosome painting demonstrated a structural rearrangement (Figure 1c), not identifiable by chromosome banding alone. In this hyperploid tumour, two elements comprised both segments of chromosome 10, and were duplicated. No normal chromosome 10 was observed. Overall, a chromosome 10 deficiency was demonstrated in 10 out of 12 tumours studied by both chromosome banding and painting, and in two other tumours studied by chromosome banding alone.

*Amount of HK-I mRNA*

HK-I mRNA amounts in tumour extracts were semiquantified by Northern blot analysis and were compared with that of GADPH, used as an internal marker. To ensure that mRNA quantification could be performed under our experimental conditions, increasing concentrations (2.5–20 $\mu$g) of TG-8-OZ extract were electrophoresed, then blotted, hybridised with the [$^{32}$P]HK-I probe and the radioactive signal measured by a phosphorimager. A linear correlation was obtained between the area under the curve and the amount of HK-I mRNA ($\alpha < 0.01$, data not shown). The individual HK-I mRNA amount in the 12 gliomas tested ranged from 0.78 to 0.28 AU, whereas HK-I mRNA expression in a normal human brain extract was 1.2. HK-I mRNA was considered as decreased when below 0.6, i.e. less than 50% of the control. Compared with normal human brain, the HK-I mRNA transcript level was drastically decreased in seven gliomas and somewhat less in the five others. The two gliomas that retained their chromosomes 10 (TG-18-CH and TG-8-OZ) had more transcripts (0.78 and

**Figure 1** Fluorescence *in situ* hybridisation on metaphase chromosomes using human total genomic biotin-labelled painting probes of chromosome 10 on three xenografted gliomas. (**a**) Specific painting of TG-15-HA xenograft, a diploid clone, revealed only one chromosome 10. (**b**) Specific painting of euploid TG-18-CH xenograft detected two chromosomes 10. (**c**) Specific painting of TG-23-GI xenograft showed a rearrangement of chromosome 10.

0.77 respectively) than the others but still only 65% of the normal brain control. The seven gliomas with low HK-I mRNA amounts were chromosome 10 deficient. Indeed, a significant correlation was found between the relative chromosome 10 number and the HK-I mRNA level ($\alpha<0.01$) (Figure 2a).

**Figure 2** (**a**) Correlation between semiquantitative HK1 mRNA in arbitrary units (AU) and relative chromosome 10 number in 15 human xenografted gliomas and normal brain tissue control. (**b**) Correlation between semiquantitative HK1 mRNA and tHK activity. (**c**) Relationships between the percent of mitochondria-bound HK and mHK activity. (**d**) Relationships between the lactate–pyruvate ratio and mHK activity. (**e**) Correlation between the lactate–pyruvate ratio and the ATP content. (**f**) Correlation between the lactate content and mHK activity.

### *HK enzymatic activity*

Total HK-I activity (expressed in mU $mg^{-1}$ protein) and its intracellular distribution were studied. Mean tHK activity was constantly lower in gliomas ($72 \pm 11$ mU) than in the normal human brain (489 mU, Table I). The intertumoral tHK activity varied widely, ranging from 17 (TG-24-RO) to 173 mU (TG-7-RO), i.e. 3.5–35% that of the control. Mean c- and mHK activates were respectively 3.4-fold and 9.6-fold lower in gliomas than in controls.

Most HK was bound to mitochondria in normal brain tissue (76%). In the majority of gliomas, HK was also mostly bound to mitochondria, except for a group of four gliomas TG-10-PY, TG-22-LU, TG-11-DU and TG-8-OZ whose mHK fractions were of 34%, 23%, 18% and 9%. Number of chromosome 10 was not correlated with HK activity as if HK decrease precedes the loss of chromosome 10. A correlation was established between tHK activity and the amount of HK-I mRNA ($\alpha < 0.05$) (Figure 2b). A significant correlation was also found between mHK fraction and mHK activity ($\alpha < 0.05$) in the xenografts (Figure 2c). This relationship was no longer significant when the normal brain value was included in the analysis.

### *Lactate–pyruvate ratio and ATP content*

Lactate and pyruvate concentrations were measured and lactate–pyruvate ratios were calculated for 13 gliomas (Table I). The lactate–pyruvate ratio was 5 in control brain. The intertumoral variations ranged from 5 to 27 (mean $16 \pm 2$). Except for TG-21-LA, this ratio was always higher in xenografted tissues than in control brain, with a mean increase of 5-fold. The higher lactate–pyruvate ratio was independent of mHK activity (Figure 2d). The ATP content (expressed in nmol $g^{-1}$ protein) was always higher in gliomas in which, despite wide interindividual variations, a mean of 50 was measured compared with 10 in the control brain. The higher ATP content was significantly correlated with the lactate–pyruvate ratio (Figure 2e) ($\alpha < 0.05$) and mHK activity when value for normal brain was not included (Figure 2f) ($\alpha < 0.05$).

### *L-Lactate dehydrogenase activity*

LDH was included as a positive enzyme control. No significant differences were observed in LDH activity in human xenografted gliomas compared with normal human brain tissue.

### Discussion

Chromosome 10 loss has been described as a recurrent event in advanced gliomas (Fujimoto *et al.*, 1989; Watanabe *et al.*, 1990; Bondy *et al.*, 1994). In our series of human xenografted gliomas, homogeneous for histology and grading, cytogenetic analyses with R-banding and chromosome painting confirmed the previous reports: chromosome 10 deficiency was detected in 10 of the 12 gliomas studied. This loss is paradoxical in these tumours which use glucose as their main source of energy metabolism (Parry and Pedersen, 1990). To maintain a highlevel of glycolysis, we postulated that gliomas either increased *HK-I* gene transcription or increased HK enzymatic activity. We attempted here to elucidate how gliomas adapt their glucose metabolism to chromosome 10 deficiency.

Despite the limitations of semiquantification using the Northern blotting technique, considerable differences between HK-I mRNA amounts in normal control brain and gliomas with or without a chromosome 10 deficit were measured. Quantification by RT-PCR would have been more quantitative; however, this technique was not performed because we had already found an important and reproducible decrease in HK-I mRNA by Northern blot in the whole glioma series. A significant correlation was found between the relative chromosome 10 number and the HK-I mRNA amount, which suggests a gene dosage effect, as proposed by Lasserre *et al.* (1994).

An alternative hypothesis to explain the decreased amount of RNA, besides less HK-I mRNA transcription, would be a more rapid degradation of the messenger RNA (shortening its half-life). As a 50% decrease in HKI-mRNA was associated with a recurrent loss of one copy of chromosome 10, no evaluation of HK-I mRNA increased degradation rate was investigated. A multistep mechanism, involving chromosome loss and altered transcription, has previously been proposed for superoxide dismutase II and catalase activities in SV-40-transformed fibroblasts (Bravard *et al.*, 1992; Hoffschir *et al.*, 1993), hormone receptors in breast cancer (Magdelénat *et al.*, 1994) and regulation of thymidylate synthase and thymidine kinase syntheses in colorectal cancer (Bardot *et al.*, 1991; Lasserre, 1994).

Decreased HK-I activity accentuates the paradox of fewer HK-I transcripts (about a third) in highly glycolytic tumours. It must be argued that HK activity measured by the technique used in the samples could detect HK-I, -II and -III. These two isoenzymes (HK-II and -III) are of minor importance in brain tissue. In addition, the gene dosage of HK-I pleads for a lack of compensation by HK-II and -III increased activity.

It is common knowledge that quantitative relationships between transcription levels of enzymes and their activities are not directly reliable. However, Fanciulli *et al.* (1994) showed that transfection of NIH-3T3 cells with a HK cDNA-induced tumour increased HK synthesis and the activity of the particulate cellular fraction 2-fold, raised the glycolysis rate and accelerated growth rate.

In the mostly grade IV xenografted gliomas studied here, the tHK activity was a mean 6.8-fold lower than that of normal human brain tissue. Floridi *et al.* (1989) also showed that tHK activity was lower in tumours than in normal brain. In our series, both c- and mHK activities were lower in gliomas than in control brain tissue, but to a lesser degree for the latter. Thus, HK remained preferentially bound to mitochondria in most of these xenografted tumours. In 11 of them, the mHK fraction represented more than 50% of the tHK activity, whereas it was below 35% in the other four. LDH activity, used as positive enzyme control, remained stable in the xenografted gliomas compared with normal brain tissue, and demonstrated that the gliomas examined have selectively adapted their HK activity.

The elevated lactate–pyruvate ratio in the large majority of the gliomas demonstrated a metabolic deviation. This increased glycolysis was not dependent on t-, c-, or mHK activity or mHK binding. Graham *et al.* (1985) showed that tumoral mHK exhibited different kinetics, with an increased maximal velocity of HK activity in glioblastoma cell lines.

High glycolysis was associated with an elevated ATP content. These results are in agreement with data showing a high rate of glycolysis in brain tumours (Floridi, 1989; Macbeth and Bekesi, 1962). Significantly lower tHK activity associated with increased ATP pools was previously described in a series of 15 human gliomas (Lowry *et al.*, 1983). Anchorage of HK-I to mitochondrial porins favours its access to ATP synthesised via oxidative phosphorylation in the inner mitochondrial compartment (Arora and Pedersen, 1988; Laterveer *et al.*, 1994). HK-I anchorage to mitochondrial porins could maintain them in 'open state', which enables a continuous ATP flux for glucose phosphorylation (McCabe, 1994). In addition, the HK-I anchorage could prevent mHK from proteolysis and, so doing, increases enzymatic protein half-life. Mitochondrial HK can assure rapid and efficient generation of ADP, which is essential for oxidative phosphorylation (BeltrandelRio and Wilson, 1991) and, as such, is less down-regulated than cHK by G-6-P. The HK-I balance between cytosolic and mitochondrial forms depends on intracellular pH ($pH_i$) (Miccoli *et al.*, 1996). A relative alkaline $pH_i$ is observed in gliomas as compared with human brain, enhancing HK binding to mitochondria. In addition, low feedback regulation of tumoral mHK and its quick transphosphorylation turnover could explain the high rate of glycolysis despite decreased c-, m- and t-HK activities. Nevertheless, other mechanims might operate in glioma cells to explain this high glycolysis. A decreased pool of mitochondria as observed in all the gliomas studied, as previously shown by Pedersen in highly glycolytic tumours, could participate in this metabolic deviation (Pedersen, 1978). Moreover HK-I responsible for initiating glycolysis may approach its lowest limit in tumours, explaining the responsiveness of xenografted gliomas to lonidamine *in vivo* (Oudard, 1995). Disrupting the HK-I mitochondria binding could constitute a new strategy for reducing glycolysis without salvage by oxidative phosphorylation.

In conclusion, this study confirms the recurrent chromosome 10 loss in advanced gliomas. The elevated glycolysis measured in these xenografted tumours cannot be explained either by adaptation of the HK-I mRNA amount or HK-I enzymatic activity. This increased level of glycolysis strongly indicates that glucose remains the major source of energy in gliomas. Paradoxically, a deficit in HK-I, one of the main steps in the metabolic chain of glucose-utilising metabolism has been demonstrated. Although this sharp drop in the activity of this key enzyme might precede the loss of chromosome 10, another phenomenon is possibly at work in reflecting a metabolic differential between normal and tumoral tissue.

**Abbreviations**

HK-I, hexokinase type I isoenzyme; t-, c- and mHK total, cytosolic, mitochondria-bound hexokinase respectively; G-6-P, glucose 6-phosphate; GAPDH, glyceraldehyde-3-phosphate-dehydrogenase; LDH, lactate dehydrogenase; SCC, saline sodium citrate; AU, arbitrary units; GBM, glioblastoma multiforme.

**Acknowledgements**

We thank Drs D Ferbus and H Magdelénat for their help and support in the realisation of this work. We are grateful to Mrs J Jacobson for reviewing the language used in this report. This work was supported by the Institut National de la Santé et de la Recherche Médicale, the Association pour la Recherche sur le Cancer and the Association pour la Recherche sur les Tumeurs Cérébrales and the Ligue contre le Cancer (Comité de la Moselle).

### References

ARORA KK AND PEDERSEN PL. (1988). Functional significance of mitochondrial bound hexokinase in tumor cell metabolism: evidence for preferential phosphorylation of glucose by intramitochondrially generated ATP. *J. Biol. Chem.*, **263**, 17422–17428.

BARDOT V, LUCCIONI C, LEFRANCOIS D, MULERIS M AND DUTRILLAUX B. (1991). Activity of thymidylate synthetase, thymidine kinase and galactokinase in primary and xenografted human colorectal cancers in relation to their chromosomal patterns. *Int. J. Cancer*, **47**, 670–674.

BELTRANDELRIO H AND WILSON JE. (1991). Hexokinase of rat brain mitochondria: relative importance of adenylate kinase and oxidative phosphorylation as source of substrate ATP, and interaction with intramitochondrial compartments of ATP and ADP. *Arch. Biochem. Biophys.*, **286**, 183–194.

BIGNER S AND MARK J. (1984). Chromosomes and chromosomal progression of human gliomas in vivo, in vitro and in athymic nude mice. *Prog. Exp. Tumor Res.*, **27**, 67–82.

BIGNER SH, MARK J, BURGER PC, MAHALEY MS, BULLARD DE, MUHLBAIER LH AND BIGNER DD. (1988). Specific chromosomal abnormalities in malignant human gliomas. *Cancer Res.*, **48**, 405–411.

BIGNER SH, MARK J AND BIGNER DD. (1990). Cytogenetics of human brain tumors. *Cancer Genet. Cytogenet.*, **47**, 141–154.

BONDY M, WIENCKE J, WRENSCH M AND KYRISTSIS AP. (1994). Genetics of primary brain tumors: a review. *J. Neurooncol.*, **18**, 69–81.

BRAVARD A, HOFFSCHIR F, SABATIER L, RICOUL M, PINTON A, CASSINGENA R, ESTRADE S, LUCCIONI C AND DUTRILLAUX B. (1992). Early superoxide dismutase alterations during SV40-transformation of human fibroblasts. *Int. J. Cancer*, **52**, 797–801.

BURGER PC. (1986). Malignant astrocytic neoplasms: classification, pathologic anatomy, and response to treatment. *Semin. Oncol.*, **13**, 16–26.

CHIGWIN JM, PRZYBYLA AE, MACDONALD RJ AND RUTTER W. (1979). Isolation of biologically active ribonucleic acid from sources enriched in ribonuclease. *Biochemistry*, **18**, 5294–5299.

FANCIULLI M, PAGGI MG, BRUNO T, CARLO CD, BONETTO F, GENTILE FP AND FLORIDI A. (1994). Glycolysis and growth rate in normal and in hexokinase-transfected NIH-3T3 cells. *Oncol. Res.*, **6**, 405–409.

FLORIDI A, PAGGI MG AND FANCIULLI M. (1989). Modulation of glycolysis in neuroepithelial tumors. *J. Neurosurg. Sci.*, **33**, 55–64.

FORT P, MARTY L, PIECHACZYK M, SABROUTY E, DANI C, JEANTEUR P AND BLANCHARD J. (1985). Various rat adult tissues express only one major mRNA species from the glyceraldehyde-3-phosphate-dehydrogenase multigenic family. *Nucleic Acids Res.*, **13**, 1431–1442.

FUJIMOTO M, FULTS DW, THOMAS GA, NAKAMURA Y, HEILBRUN MP, WHITE R, STORY JL, NAYLOR SL, KAGAN-HALLET KS AND SHERIDAN PJ. (1989). Loss of heterozygosity on chromosome 10 in human glioblastoma multiforme. *Genomics*, **4**, 210–214

GRAHAM JF, CUMMINS CJ, SMITH BH AND KORNBLITH PL. (1985). Regulation of hexokinase in cultured gliomas. *Neurosurgery*, **17**, 537–542.

HOFFSCHIR F, VUILLAUME M, SABATIER L, RICOUL M, DAYA-GROSJEAN L, ESTRADE S, CASSINGENA R, CALVAYRAC R, SARASIN A AND DUTRILLAUX B. (1993). Decrease in catalase activity and loss of the 11p chromosome arm in the course of SV40 transformation of human fibroblasts. *Carcinogenesis*, **14**, 1569–1572.

JAMES CD, CARLBOM E, DUMANSKI JP, HANSEN M, NORDENSKJOLD M, COLLINS VP AND CAVENEE WK. (1988). Clonal genomic alterations in glioma malignancy stages. *Cancer Res.*, **48**, 5546–5551.

LASSERRE C, SABATIER L, BEAUMATIN J, LUCCIONI C, LEFRANCOIS D, MULLERIS M AND DUTRILLAUX B. (1994). Gene dosage and expression, and enzyme activity of thymidine kinase and thymidylate synthase in xenografted colorectal adenocarcinomas. *Int. J. Cancer*, **56**, 1–6.

LATERVEER FD, HEIJDEN RVD, TOONEN M AND NICOLAY K. (1994). The kinetic consequences of binding of hexokinase-I to the mitochondrial outer membrane. *Biochim. Biophys. Acta*, **1188**, 251–259.

LOWRY OH, BERGER SJ, CARTER JG, CHI MMY, MANCHESTER JK, KNOR J AND PUSATERI ME. (1983). Diversity of metabolic patterns in human brain tumors: enzymes of energy metabolism and related metabolites and cofactors. *J. Neurochem.*, **41**, 994–1010.

LUNDIN A, HASENSON M, PERSSON J AND POUSETTE A. (1986). Estimation of biomass in growing cell lines by adenosine triphosphate assay. *Methods Enzymol.*, **113B**, 27–42

MACBETH RAL AND BEKESI JG. (1962). Oxygen consumption and anaerobic glycolysis of human malignant and normal tissue. *Cancer Res.*, **22**, 244–248.

MCCABE ERB. (1994). Microcompartmentation of energy metabolism at the outer mitochondrial membrane: role in diabetes mellitus and other diseases. *J. Bioenerg. Biomembr.*, **26**, 307–315.

MAGDELÉNAT H, GERBAULT-SEUREAU M AND DUTRILLAUX B. (1994). Relationship between loss of estrogen and progesterone receptor expression and of 6q and 11q chromosome arms in breast cancer. *Int. J. Cancer*, **57**, 63–66.

MANIATIS T, FRITCH E AND SAMBROOK J. (1982). *Molecular Cloning: A Laboratory Manual.* Cold Spring Harbor Laboratory Press: Cold Spring Harbor, New York.

MICCOLI L, OUDARD S, SUREAU F, POIRSON F, DUTRILLAUX B AND POUPON MF. (1996). Intracellular pH governs the subcellular distribution of hexokinase in a glioma cell line. *Biochem. J.*, **313**, 957–962.

NISHI S, SEINO S AND BEL GI. (1988). Human hexokinase: sequences of amino- and carboxyl-terminal halves are homologous. *Biochem. Biophys. Res. Comm.*, **157**, 937–943.

OUDARD S, POIRSON F, MICCOLI L, BOURGEOIS Y, VASSAULT A, POISSON M, MAGDELÉNAT H, DUTRILLAUX B AND POUPON MF. (1995). Mitochondria-bound hexokinase as target for therapy of malignant gliomas. *Int. J. Cancer*, **62**, 216–222.

PARRY DM AND PEDERSEN PL. (1990). Glucose catabolism in brain. *J. Biol. Chem.*, **265**, 1059–1066.

PEDERSEN PL. (1978). Tumor mitochondria and the bioenergetics of cancer cells. *Prog. Exp. Tumor Res.*, **22**, 190–274.

SCHWARTZ D. (1994). *Méthodes Statistiques à l'Usage des Médecins et des Biologistes*, 4th edn, Medecine-Sciences Flammarion: Paris.

SHOWS TB, EDDY RL, BYERS MG, HALEY LL, HENRY WM, NISHI S AND BELL GI. (1989). Localization of the human hexokinase I gene (HK1) to chromosome 10q22. *Cytogenet. Cell. Genet.*, **51**, 1079.

SOKOLOFF L, REIVICH M, KENNEDY C, DESROSIERS MH, PATLAK CS, PETTIGREW D, SAKURADA O AND SHINOHARA M. (1977). The [$^{14}$C]deoxyglucose method for the measurement of local cerebral glucose utilization: theory, procedure and normal values in conscious and anesthetized albino rats. *J. Neurochem.*, **28**, 897–916.

SPRENGERS ED, KOENDERMAN AHL AND STAAL GEJ. (1983). Mitochondrial and cytosolic hexokinase from rat brain: one and the same enzyme? *Biochim. Biophys. Acta*, **755**, 112–118.

STECK PA, LIGON AH, CHEONG P, YUNG WKA AND PERSHOUSE MA. (1995). Two tumor-suppressive loci on chromosome 10 involved in human glioblastomas. *Genes Chrom. Cancer*, **12**, 255–261.

VASSAULT A, BONNEFONT JP, SPECOLA N AND SAUDUBRAY JM. (1991). Lactate, pyruvate and ketone bodies. In *Techniques in Diagnostic Human Biochemical Genetics, a Laboratory Manual.* Hommes SA (ed) pp. 285–308. Wiley-Liss: New York.

WARBURG O. (1956). On the origin of cancer cells. *Science*, **123**, 309–314.

WATANABE K, NAGAI M AND WAKAI S. (1990). Loss of constitutional heterozygosity in chromosome 10 in human glioblastoma. *Acta Neuropathol.*, **80**, 251–254.

WILSON JE. (1995). Hexokinases. *Rev. Physiol. Biochem. Pharmacol.*, **126**, 65–198.